# Knowledge and use of the international classification of functioning, disability and health: a cross-sectional survey among health professionals in Pakistan

**DOI:** 10.3389/fresc.2026.1786216

**Published:** 2026-05-25

**Authors:** Muhammad Kashif Aakash, Marianna Capecci, Francesca Gimigliano, Maria Gabriella Ceravolo

**Affiliations:** 1 Department of Mental and Physical Health and Preventive Medicine, University of Campania, Luigi Vanvitelli, Naples, Italy; 2 Department of Experimental and Clinical Medicine, Marche Polytechnic University UNIVPM, Ancona, Italy

**Keywords:** disability assessment, health professionals, ICF (International classification of functioning, disability and health), Pakistan, rehabilitation

## Abstract

**Background:**

The International Classification of Functioning, Disability and Health (ICF), developed by the World Health Organization (WHO), provides a standardized framework for understanding and measuring health and disability. While widely adopted globally, its implementation in low- and middle-income countries (LMICs), such as Pakistan, remains inconsistent and underexplored.

**Objective:**

This study aimed to assess the knowledge and practical application of the ICF among healthcare professionals in Pakistan.

**Methods:**

A cross-sectional online survey was conducted using the Kobo Toolbox platform and distributed via social media to a diverse group of healthcare professionals. The survey comprised closed-ended questions across three domains: (I) demographics, education, and professional experience; (II) knowledge and utilisation of the ICF in clinical practice; and (III) clinical practice settings. A filtering question determined whether respondents claimed familiarity with the ICF. Actual knowledge was further evaluated through a four-item multiple-choice instrument; respondents answering at least two questions correctly were operationally classified as having moderate to good knowledge.

**Results:**

Of the 52 respondents, 63.5% held a medical degree—including two (6.1%) with training in Physical and Rehabilitation Medicine—while 36.5% were non-medical healthcare professionals (including nurses and medical students). While 48% claimed familiarity with the ICF, only 17% demonstrated actual moderate to good knowledge upon evaluation. ICF literacy was more prevalent among those holding doctoral degrees or at least 16 years of professional experience.

**Conclusion:**

These findings reveal an apparent gap between perceived and actual ICF knowledge among health professionals in Pakistan. The integration of ICF content into medical curricula, professional training, and continuing medical education (CME) is recommended to enhance disability assessment and rehabilitation planning in Pakistan.

## Background

In 2001, the World Health Organization (WHO) introduced the International Classification of Functioning, Disability and Health (ICF) as a comprehensive framework for describing and assessing health and disability.

Unlike the traditional biomedical model, the ICF adopts a biopsychosocial approach, emphasizing the interaction between an individual's health condition, personal attributes, and environmental factors (1). This multidimensional model enables more holistic documentation of functioning and disability, supporting clinical decision-making, research, policy formulation, and health data collection (2).

Two key global developments have reinforced the ICF's significance. First, the increasing demand for robust epidemiological data on disability has made standardized frameworks like the ICF essential for informing public health policy (3).

Second, the adoption of the ICF model in the 2006 United Nations Convention on the Rights of Persons with Disabilities (UNCRPD) has shifted the understanding of disability from a purely medical condition to a context-dependent experience, reinforcing the ICF value in promoting inclusive practices (4).

In recent years, international rehabilitation and policy bodies have made significant efforts to integrate the ICF into clinical and administrative practice. For example, the Chinese Association of Physical Rehabilitation Medicine (CARM) has developed a standardized ICF Clinical Assessment Tool, with support from the International Society of Physical and Rehabilitation Medicine (ISPRM) and other regional societies to promote its global implementation (5). These tools aim to facilitate daily clinical use, enable standardized functional reporting, and guide evidence-based policy decisions (6).

The ISPRM has also proposed a detailed policy agenda and implementation plan advocating for ICF-based reporting in national quality assurance programs, the development of clinical assessment tools, and data collection across multiple languages (6). A user-friendly mobile application in China further demonstrates how ICF tools can be embedded in routine clinical and rehabilitation workflows (7).

The ICF has shown practical utility across a range of healthcare applications. In cardiac rehabilitation (8), it enables a comprehensive assessment of functional outcomes and fosters interdisciplinary collaboration through its standardized coding system. It has also proven valuable in post-acute geriatric rehabilitation (9), the assessment of people with Parkinson's disease (10), and stroke rehabilitation (11, 12), have emphasized the need for gerontologists to adopt the ICF as a common language in disablement research.

Clinically, the ICF has been used to highlight the role of environmental factors in acquired brain injury (13), assess health status in individuals with HIV/AIDS (14), and document functioning in children and adults with complex conditions (15, 16).

In the field of occupational therapy, awareness and application of the ICF have increased, largely due to the advocacy efforts of the World Federation of Occupational Therapists (WFOT) (17).

Despite global advances, gaps in knowledge and use of the ICF remain. A survey among athletic trainers revealed inconsistent application of the framework in sports settings, with many practitioners unaware of how to implement it effectively (18). Similarly, the American Speech-Language-Hearing Association (ASHA) has adopted the ICF to guide its “Person-Centered Focus on Function” series, which addresses conditions like dementia and traumatic brain injury (3).

In Sweden, the ICF has been integrated into electronic health records to improve structured documentation in elderly care services (19).

Educational institutions worldwide, including in South Africa, are incorporating the ICF into curricula to promote its use among future healthcare professionals (20).

However, studies still show poor knowledge and limited assessment integration in some physical therapy programs (21, 22).

Beyond clinical and educational use, the ICF supports administrative and research functions. It is employed in data aggregation by interdisciplinary health teams and as a framework for enhancing interprofessional education (16).

With the rise of artificial intelligence, there is growing interest in using AI tools to automate ICF coding, identify trends in functional outcomes, and generate personalized functional profiles based on health and environmental data (23).

Globally, many healthcare decision-makers now regard the ICF as the most valid and reliable standard for capturing functioning and disability (24); yet many health professionals continue to rely on traditional diagnostic models.

In particular, the extent of ICF knowledge, usage, and integration within healthcare systems in low- and middle-income countries (LMIC) —including Pakistan—remains unclear (25, 26).

## Objectives

The primary objective of this study is to evaluate the level of knowledge and practical use of the ICF among healthcare professionals in Pakistan.

A secondary objective is to identify demographic and professional factors associated with ICF implementation across various clinical settings.

## Research methodology

### Study design

In this cross-sectional study, we examined the knowledge and use of the ICF in health professionals in Pakistan. A close-ended questionnaire was designed by the 2nd, 3rd and 4th authors of the paper, who are professors and PRM Physicians having expertise in rehabilitation medicine.

The questionnaire was subsequently configured as an anonymous online survey with Kobo Toolbox by the corresponding author. The survey consisted of 25 closed-ended questions (Supplementary Material / Appendix 1) and was designed to assess three domains: (I) demographic information, education, and professional experience (questions 1–7); (II) knowledge and use of the ICF in clinical practice (questions 8–16); and (III) clinical practice settings (questions 17–25).The (Tables 1 and 2) further provide the demographic and clinic practices and settings details respectively.

**Table 1:** Education and professional experience of respondents.

S. No.	Title	Categories	N (%)
1.	**Education**	Bachelor's Degree	28 (53.85%)
		Master's Degree	15 (28.85%)
		Doctoral Degree	9 (17.31%)
2.	**Medical Degree**	Yes	33 (63.46%)
		No	19 (36.54%)
3.	**Medical Specialty**	Internal Medicine	8 (24.24%)
		Neurology	2 (6.06%)
		Physical and Rehabilitation Medicine	2 (6.06%)
		General Medicine	9 (27.27%)
		General Surgery	10 (30.30%)
		Public Health	1 (3.03%)
		Virology and Immunology	1 (3.03%)
4.	**Experience**	Less than 2 years	10 (19.23%)
		2–5 years	27 (51.92%)
		6–10 years	8 (15.38%)
		11–15 years	1 (1.92%)
		16–20 years	4 (7.69%)
		More than 20 years	2 (3.85%)
5.	**Workplace**	Hospital	27 (51.92%)
		Outpatient Rehabilitation	3 (5.77%)
		Primary Healthcare Unit	1 (1.92%)
		University	8 (15.38%)
		University Hospital	13 (25.00%)
6.	**Location**	Balochistan	9 (17.31%)
		Gilgit Baltistan	1 (1.92%)
		Islamabad	2 (3.85%)
		Khyber Pakhtunkhwa	30 (57.69%)
		Punjab	4 (7.69%)
		Sindh	6 (11.54%)
7.	**Duty Station**	Rural	9 (17.31%)
		Urban	43 (82.69%)

**Table 2:** Clinical practice and settings.

S. No.	Title	Categories	N (%)
**Disability and Rehabilitation**			
1.	**What kind of disabled patients receive treatment in your clinic, hospital?**	Cardiopulmonary	4 (7.69%)
		Mental	6 (11.54%)
		Musculoskeletal	12 (23.08%)
		Neurodevelopmental	1 (1.92%)
		Neurological	10 (19.23%)
		Oncological	1 (1.92%)
		Oncological, Neurological, Neurodevelopmental, Cardiopulmonary, Musculoskeletal, Mental	8 (15.38%)
		Others	10 (19.23%)
2.	**What kind of disability do your patients mostly present?**	Acute Disability	36 (69.23%)
		Chronic Disability	16 (30.77%)
3.	**Rehabilitation availability in hospitals/clinics**	Home Rehabilitation	5 (22.73%)
		Rehabilitation in acute hospital	6 (27.27%)
		Outpatient rehabilitation	4 (18.18%)
		Specialist Rehabilitation ward (i.e., Spinal Cord injury or Brain injury unit);	2 (9.09%)
		Rehabilitation in acute hospital, Intensive inpatient rehabilitation, Specialist Rehabilitation ward (i.e., Spinal Cord injury or Brain injury unit), Outpatient rehabilitation	3 (13.64%)
		Intensive inpatient rehabilitation	2 (9.09%)
4.	**How many treatment hours/days patients receive in outpatient rehabilitation**	less than 1 h	16 (30.77%)
		1–2 h	21 (40.38%)
		3–5 h/day or more	15 (28.85%)
5.	**How many treatment hours/day patients receive in intensive rehabilitation unit**	Less than 1 h	17 (32.69%)
		1–2 h	17 (32.69%)
		3 h/day or more	18 (34.62%)
6.	**Is technology-aided rehabilitation used according to your experience?**	Yes	19 (36.54%)
		No	33 (63.46%)
7.	**What kind of technology?**	Non-invasive brain stimulation	3 (15.79%)
		Robotic rehabilitation	6 (31.58%)
		Tele rehabilitation with telemonitoring	1 (5.26%)
		Virtual reality rehabilitation	6 (31.58%)
		Robotic rehabilitation, Non-invasive brain stimulation, Tele rehabilitation with telemonitoring	3 (15.79%)

A filtering question asked respondents whether they were familiar with the ICF. Those who responded affirmatively answered four multiple-choice questions designed to assess their ICF knowledge. Respondents who answered at least two of the four questions correctly were categorized as having “moderate to good knowledge”; those who answered one or none correctly, or did not answer at all, were classified as having “poor knowledge.”

The four knowledge assessment items were developed by the second, third, and fourth authors, all of whom are Physical and Rehabilitation Medicine physicians with expertise in ICF clinical application and education. Each item was designed to target a distinct ICF component: Q1 assessed activity limitation, Q2 assessed body function impairment, Q3 assessed understanding of the performance qualifier, and Q4 assessed knowledge of environmental factors. The items were reviewed iteratively by the full author team for content clarity and alignment with WHO ICF documentation. No formal pilot testing or independent psychometric validation was conducted prior to administration, which is acknowledged as a limitation. The threshold of at least two correct responses out of four (≥50%) was established as a pragmatic minimum competency benchmark, consistent with binary classification approaches used in similar ICF knowledge surveys in low- and middle-income countries (27, 28).

A convenience sampling strategy was used to recruit the participant of the study. The survey was disseminated via social media platforms (Facebook and LinkedIn), accompanied by a breif introductory text targeting healthcare professionals across various disciplines and levels of training. The participant self-selected by voluntarily completing the online questionnaire.

Participation was limited to one submission per respondent. Ethical considerations, including participant consent and confidentiality, were maintained throughout. Data collection was conducted over approximately 40 days from February to March 2024.

The reporting of this study adheres to the STROBE (Strengthening the Reporting of Observational Studies in Epidemiology) statement for cross-sectional studies (29).

### Data analysis

Survey data were exported from Kobo Toolbox into Microsoft Excel; the exported raw data were cleaned and subsequently imported into Stata software for analysis. Descriptive statistics were used to summarize participant demographics and responses, and missing responses were coded as such.

To assess knowledge, respondents who correctly answered at least two of the four ICF-related questions were deemed to have “moderate to good knowledge.” In contrast, others were considered to have “poor knowledge.”.

Summary statistics were then used to compare claimed knowledge vs. actual knowledge. Fisher's Exact Test was applied to explore associations between ICF knowledge (both claimed and actual) and key demographic and professional variables: education, medical specialty, years of experience, workplace type, region, and duty station (urban vs. rural). Due to the small sample size and categorical nature of the data, Fisher's Exact Test was deemed appropriate. It should be noted that the study was not formally powered to detect statistically significant associations across subgroups, and all inferential comparisons should therefore be interpreted with caution.

## Results

### Participants

A total of 52 healthcare professionals responded to the survey. Participants were drawn from six regions in Pakistan: Khyber Pakhtunkhwa, Balochistan, Punjab, Sindh, Gilgit Baltistan, and Islamabad. Education levels were as follows: 53.85% held a bachelor's degree, 28.85% a master's degree, and 17.31% a doctoral degree. A majority (63.46%) had a medical background.

The most common specialties were general surgery (30.3%), general medicine (27.27%), and internal medicine (24.24%). Over half (51.92%) had 2–5 years of work experience. Most respondents worked in urban areas (82.69%), primarily in hospitals (51.92%) or university hospitals (25%). Khyber Pakhtunkhwa and Balochistan accounted for the highest response rates.

### Claimed knowledge and Use of ICF

Among respondents, 48.08% claimed to know the ICF, while 51.92% reported no awareness. Of those claiming knowledge, 56.52% indicated they used the ICF in practice, primarily in clinical (53%) and research (23%) settings. However, 43.48% admitted not using it despite claiming familiarity with the tool.

### Actual knowledge of ICF

Respondents who claimed to know the ICF (*n* = 25) were asked four multiple-choice questions. Of the 23 who answered, only 9 (39.13%) demonstrated moderate to good knowledge; the remaining 14 (60.86%) had poor knowledge. Two respondents did not answer and were also categorized as having poor knowledge.

Based on these findings, we categorized respondents in two groups: ICF Literate: 9 respondents (17.30% of total sample) and ICF Unaware: 43 respondents (82.69% of total sample).

Using Fisher's Exact Test to examine relationships between ICF knowledge (claimed and actual) and other recorded variables, no statistically significant associations were identified between ICF knowledge and education level, medical degree, work experience, workplace, geographic region, and urban vs. rural duty station (Tables 3–8).

**Table 3:** ICF knowledge by education level.

Do you know ICF	Education			Total
	Bachelor's Degree	Master's Degree	Doctoral Degree	
No	16	8	3	27
	(59.26%)	(29.63%)	(11.11%)	(100%)
	{57.14%}	{53.33%}	{33.33%}	{51.92%}
Yes	12	7	6	25
	(48%)	(28%)	(24%)	(100%)
	{42.86%}	{46.67%}	{66.67%}	{48.08%}
Total	28	15	9	52
	(53%)	(28.85%)	(17%)	(100%)
	{100%)	{100%}	{100%}	{100%}

**Fisher exact** = 0.523.

() Row percentages, {} Column Percentages.

**Table 4:** ICF knowledge by medical specialization.

Do you know ICF	Medical Degree							
	Internal Medicine	Neurology	Physical and Rehabilitation Medicine	General Medicine	General Surgery	Public Health	Virology and Immunology	Total
No	4	0	0	6	5	1	0	16
	(25%)	0	0	(37.25%)	(31.25%)	(6%)	0	(100%)
	{50%}	0	0	{50%}	{50%}	{100%)	0	{48.48%}
Yes	4	2	2	3	5	0	1	17
	(23.53%)	(11.76%)	(11.76%)	(17.65%)	(29%)	0	(5.88%)	(100%)
	{50%}	{100%}	{100%)	{33.33%}	{50%}	0	{100%}	{51.52%)
Total	8	2	2	9	10	1	1	33
	(24.24%)	(6.06%)	(6.06%)	(27.27%)	(30%)	(3%)	(3.03%	(100%)
	{100}	{100%)	{100%}	{100%}	{100%}	{100%}	{100%}	{100%}

**Fisher exact** = 0.402.

() Row percentages, {} Column Percentages.

**Table 5:** ICF knowledge by workplace.

Do you know ICF	Workplace					
	Hospital	Outpatient Rehabilitation	Primary Healthcare Unit	University	University Hospital	Total
No	14	1	0	4	8	27
	(51.85%)	(3%)	0	(14.81%)	(29%)	(100%)
	{51.85%}	{33%}	0	{50%}	{61%}	{5,192%}
Yes	13	2	1	4	5	25
	(52%)	(8%)	(4%)	(16%)	(20%)	(100%)
	{48%}	{66%}	{100%}	{50%}	{38%}	{48.08%}
Total	27	3	1	8	13	52
	(51%)	(5%)	(1.92%)	(15%)	(25%)	(100%)
	{100%}	{100%}	{100%)	{100%}	{100%}	{100%}

**Fisher exact** = 0.815.

() Row percentages, {} Column Percentages.

**Table 6:** ICF knowledge by work experience.

Do you know ICF?	Work Experience						
	Less than 2 years	2–5 Years	6–10 Years	11–15 Years	16–20 Years	More than 20 Years	Total
No	5	14	6	0	1	1	27
	(18.52%)	(51.85%)	(22.22%)	0	(3.70%)	(3.70%)	(100%)
	{50%}	{51.85%}	{75%}	0	{25%}	{50%}	{51.92%}
Yes	5	13	2	1	3	1	25
	(20%)	(52%)	(8%)	(4%)	(12%)	(4%)	(100%)
	{50%}	{48.15%}	{25%}	{100%}	{75%}	{50%}	{48.08%}
Total	10	27	8	1	4	2	52
	(19.23%)	(51.92%)	(15.38%)	(1.92%)	(7.69%)	(3.85%)	(100%)
	{100%}	{100%}	{100%}	{{100%}	{100%}	{100%}	{100%}

**Fisher exact** = 0.594.

() Row percentages, {} Column Percentages.

**Table 7:** ICF knowledge by location.

Do you know ICF?	Location						
	Balochistan	Gilgit Baltistan	Islamabad	Khyber Pakhtunkhwa	Punjab	Sindh	Total
No	4	0	1	13	4	5	27
	(14.81%)	0	(3.70%)	(48%)	(14.81%)	(18.52%)	(100%)
	{44.44%}	0	{50%}	{43%}	{100%}	{83%}	{51.92%}
Yes	5	1	1	13	0	1	25
	(20%)	(4%)	(4%)	(68%)	0	(4%)	(100%)
	{55.56%}	{100%}	{50%}	{56.67%}	0	{16.67%}	{48.08%}
Total	9	1	2	30	4	6	52
	(17.31%)	(1.92%)	(3.85%)	(57.69%)	(7.69%)	(11.54%)	(100%)
	{100%}	{100%}	{100%}	{100%}	{100%}	{100%}	{100%}

**Fisher exact** = 0.103.

() Row percentages, {} Column Percentages.

**Table 8:** ICF knowledge by duty station.

Do you know ICF?	Duty Station		
	Rural Area	Urban Area	Total
No	4	23	27
	(14.81%)	(85.19%)	(100%)
	{44.44%}	{53.49%}	{51.92%}
Yes	5	20	25
	(20%)	(80%)	(100%)
	{55.56%}	{46.51%}	{48.08%}
Total	9	43	52
	(17.31%)	(82.69%)	(100%)
	{100%}	{100%}	{100%}

**Fisher exact** = 0.722.

() Row percentages, {} Column Percentages.

## Discussion

To our knowledge this is among the first studies to assess both perceived and actual knowledge of the International Classification of Functioning, Disability and Health (ICF) among healthcare professionals in Pakistan, though the exploratory nature of the study warrants cautious interpretations of the findings.

While nearly half of the respondents reported familiarity with the ICF, only 17% demonstrated adequate understanding upon evaluation. This apparent discrepancy within the surveyed sample suggests that reported awareness may, in some cases, reflect recognition of the acronym rather than a substantive understanding of the ICF principles and their clinical applications.

The distinction between self-reported familiarity and demonstrated knowledge warrants careful consideration. Self-reported familiarity is vulnerable to social desirability bias and acronym recognition — respondents may indicate awareness of the ICF because they have encountered the term, without having developed a substantive understanding of its conceptual framework or clinical utility. Demonstrated knowledge, assessed through scenario-based items, reflects a different and arguably more clinically relevant construct. The gap observed between these two measures in the current sample is consistent with findings from comparable settings, where similar knowledge gaps have been documented among rehabilitation professionals in other LMIC settings, commonly attributed to inadequate integration of ICF content into professional training programmes (27, 28). It should also be acknowledged that the four-item instrument used here captures only a narrow slice of ICF competence and should not be interpreted as a comprehensive measure of ICF literacy. Accordingly, the observed gap should be regarded as indicative rather than definitive and interpreted within the constraints of the study design.

Our findings echo broader trends observed in low- and middle-income countries (LMICs), where the ICF framework remains underutilized despite its significant potential to enhance disability assessment and foster interdisciplinary collaboration. The limited engagement with ICF concepts observed in this sample suggests a missed opportunity for healthcare systems in resource-constrained settings such as Pakistan, where efficient and holistic rehabilitation services are needed.

To date, only one prior study in Pakistan—by Jafri and Camargo (30)—has explored healthcare professionals' familiarity with the ICF. Their survey of 32 diverse health professionals (including physicians, nurses, physical therapists, occupational therapists, psychologists and speech therapists) in Karachi found that two-thirds reported some level of familiarity.

However, their methodology did not investigate actual knowledge or practical application, limiting the depth of insight. By contrast, our study extends prior work by assessing participants actual understandings, thereby offering a more nuanced picture of ICF awareness among the surveyed professionals.

Comparative international evidence appears consistent with the patterns observed in this study. In Iran, Kimiafar et al. (28) reported that rehabilitation specialists understood the general scope of the ICF but lacked deeper comprehension of its components.

Similarly, a large-scale survey in Brazil by Pernambuco et al. (27) revealed that most rehabilitation professionals (1,313 cases, 85% physiotherapists and 15% occupational therapists) did not incorporate the ICF into clinical practice due to inadequate training. The authors called for formal inclusion of ICF content in both undergraduate and professional development programs—a recommendation equally pertinent for Pakistan.

Qualitative research from other contexts supports these concerns. Peters-Brinkerhoff's case study in the U.S (22). showed that physical therapy students received little to no instruction on the ICF, and performance evaluations omitted any reference to its use.

In South Africa, Hall (31) documented a gap between theoretical ICF knowledge and its practical application, attributed to the absence of clear guidelines for healthcare providers.

Likewise, an RCT conducted by Sagahutu (32) in Rwanda demonstrated that targeted training could significantly improve professionals' understanding of the ICF, suggesting a model for effective educational interventions in similar settings.

### Study limitations

This study is subject to several limitations that warrant consideration when interpreting the findings. First, the sample comprised only 52 respondents and was recruited through convenience sampling via social media, which likely introduced selection bias toward digitally engaged, urban-based, and academically affiliated professionals; no *a priori* sample size calculation was undertaken, given the exploratory nature of the study. Second, the cohort included very few specialists in Physical and Rehabilitation Medicine — the professional group most likely to apply the ICF in routine practice — thereby constraining inferences specific to rehabilitation practitioners. Third, actual knowledge was operationalized through a four-item multiple-choice instrument, which offers limited construct coverage of the ICF framework and lacks formal psychometric validation; consequently, it captures conceptual recognition rather than applied ICF reasoning. Accordingly, the findings should be regarded as preliminary and hypothesis-generating rather than as a definitive characterization of ICF literacy among health professionals in Pakistan. To support reproducibility, the full survey instrument is provided in Supplementary Material / Appendix 1, and the open-access Kobo Toolbox link is included therein, enabling replication in comparable settings.

## Conclusion

Exploring awareness and utilisation of the ICF among healthcare professionals in Pakistan represents an important step toward identifying implementation gaps and informing educational and policy initiatives. This study identifies an important gap between perceived and actual ICF knowledge among healthcare professionals in Pakistan. Unlike previous research in Pakistan that focused solely on self-reported familiarity, this study assessed actual knowledge, offering a more objective indication of ICF awareness among the surveyed professionals. These findings support the integration of ICF content into formal medical education and continuing professional development in Pakistan. Future research employing larger, randomly sampled, and more representative cohorts is needed to substantiate these observations and evaluate the impact of structured educational interventions on ICF knowledge and clinical application.

## Data Availability

The raw data supporting the conclusions of this article will be made available by the authors, without undue reservation.
