# Supplementary material for: Knowledge and use of the international classification of functioning, disability and health: a cross-sectional survey among health professionals in Pakistan
Source: Front Rehabil Sci. 2026 May 25;7:1786216. doi: 10.3389/fresc.2026.1786216 (PMC13243374; doi:10.3389/fresc.2026.1786216)
Supplement: Supplementary file 1 [file Supplementaryfile1.docx]

Appendix 1: Survey Questionnaire

| Question Type | Questions |
| --- | --- |
| **Education and Professional Experience** | |
| 1. Multiple Choice | What is your Higher Degree? |
|  | Bachelor’s degree Master’s Degree Doctoral Degree |
| 1. Multiple Choice | How long ago did you achieve your first degree? |
|  | Less than 2 years 2-5 years 6-10 years 11-15 years 16-20 years More than 20 years |
| 1. Multiple Choice | Did you achieve a medical specialty or are you training in a medical specialty? |
|  | Yes No |
| 1. Multiple Select | What specialty do you have or are you training in? |
|  | Neurology Physical and Rehabilitation Medicine Internal Medicine Others… (Specify) |
| 1. Multiple Select | Where do you work? |
|  | University Hospital Hospital  University Primary Healthcare Unit Outpatient Rehabilitation Homecare |
| 1. Multiple Choice | Where is your duty station in Pakistan? |
|  | Khyber Pakhtunkhwa Punjab Balochistan Sindh Gilgit Baltistan Islamabad |
| 1. Multiple Choice | Describe your duty station? |
|  | Urban  Rural |
| **Knowledge and Use of ICF in Clinical Practice** | |
| 8. Multiple Choice | Do you know the ICF? |
|  | Yes  No |
| 9. Multiple Choice | What is the extended definition of the ICF? |
|  | International Physiotherapy Classification  Integrated Physical Therapy Clinics  International Classification of Functioning, Disability and Health  International Classification of Functioning, Activity and Participation |
| 10. Multiple Choice | When did you learn about ICF? |
|  | Bachelor  Specialization  Master or PhD  Clinical Practice  CME/CPD Courses |
| 11. Multiple Choice | How is "inability to walk around obstacles" in a physiotherapy room classified according to the ICF? |
|  | Body Function Impairment  Body Structure Impairment  Participation Restriction  Activity Limitation  Environmental Barrier |
| 12. Multiple Choice | How is "inability to swallow" classified according to ICF? |
|  | Body Function Impairment  Body Structure Impairment  Participation Restriction  Activity Limitation  Environmental Barrier |
| 13. Multiple Choice | What does the performance qualifier of the ICF describe? |
|  | The individual's ability to execute a task or an action  The highest probable level of functioning that a person may reach in a given domain  The lived experience of people in the actual context in which they live  The extent to which an activity is limited  The global human functioning in a standardized environment |
| 14. Multiple Choice | In which component of the ICF are prosthetic and orthotic devices classified? |
|  | Activities and Participation  Body Functions  Body Structures  Environmental Factors  Personal Factors |
| 15. Multiple Choice | Do you use ICF? |
|  | Yes  No |
| 16. Multiple Select | In Which area do you use ICF? |
|  | Clinic  Research  Teaching |
| **Clinical Practice and Settings** | |
| 17. Add comment | If you don’t use ICF, please indicate the tools you use to detect/quantify disability? |
| 18. Multiple Select | What kind of disabled patients receive treatment in your clinic, hospital? |
|  | Oncological  Neurological  Neurodevelopmental  Cardiopulmonary Musculoskeletal  Mental  Others…. Specify… |
| 19. Multiple Choice | What kind of disability do your patient mostly present? |
|  | Acute  Chronic |
| 20. Multiple Select | Which of the following rehabilitation settings are you directly involved in, if any? |
|  | Not involved  Rehabilitation in acute hospital  Intensive inpatient rehabilitation  Specialist Rehabilitation ward (i.e. Spinal Cord injury or Brain injury unit)  Outpatient rehabilitation  Home rehabilitation  Others… Specify… |
| 21. Multiple Select | If you are not directly involved in the rehabilitation process, which of the following rehabilitation settings are mostly available for your disabled patients? |
|  | Rehabilitation in acute hospital  Intensive inpatient rehabilitation  Specialist Rehabilitation ward (i.e. Spinal Cord injury or Brain injury unit)  Outpatient rehabilitation  Home rehabilitation  Others… Specify… |
| 22. Multiple Choice | How many treatment hours/day patients receive in intensive rehabilitation units? |
|  | Less than 1 hour  1-2 hours  3 hours/day or more |
| 23. Multiple Choice | How many treatment hours/week patients receive in outpatient rehabilitation services? |
|  | Less than 1 hour  1-2 hours  3 hours/day or more |
| 24. Multiple Choice | Is technology-aided rehabilitation used according to your experience? |
|  | Yes  No |
| 25. Multiple Select | What kind of technology? |
|  | Robotic Rehabilitation  Virtual Reality Rehabilitation  Non-invasive brain stimulation  Tele rehabilitation with telemonitoring |
| Questionnaire link | <https://ee.kobotoolbox.org/single/gLTT31zX> |
