# Supplementary material for: Knowledge and use of the international classification of functioning, disability and health: a cross-sectional survey among health professionals in Pakistan
Source: Front Rehabil Sci. 2026 May 25;7:1786216. doi: 10.3389/fresc.2026.1786216 (PMC13243374; doi:10.3389/fresc.2026.1786216)
Supplement: Supplementary file 2 [file Table1.docx]

Appendix 2: Knowledge and Use of ICF in Clinical Practice

| **S. No.** | **Title** | **Categories** | **N (%)** |
| --- | --- | --- | --- |
| **Knowledge of ICF** | | | |
|  | **ICF** | Yes | 25 (48.08%) |
|  |  | No | 27 (51.92%) |
|  | **ICF Acronym** | Integrated Physical Therapy Clinics | 2 (3.85%) |
|  |  | International Classification of Function, Activity and Participation | 4 (7.69%) |
|  |  | International Classification of Functioning, Disability and Health (correct) | 41 (78.85%) |
|  |  | International Physiotherapy Classification | 5 (9.62%) |
|  | **ICF.Q1. How is “inability to walk around obstacles” classified according to ICF?** | Activity Limitation (correct) | 7 (30.43%) |
|  |  | Body function impairment | 12 (52.17%) |
|  |  | Body structure impairment | 3 (13.04%) |
|  |  | Participation restriction | 1 (4.35%) |
|  | **ICF.Q2. How is "inability to swallow" classified according to ICF?** | Activity Limitation | 2 (8.70%) |
|  |  | Body function impairment (correct) | 16 (69.57%) |
|  |  | Body structure impairment | 5 (21.74%) |
|  | **ICF.Q3. What does the performance qualifier of the ICF describe?** | The extent to which an activity is limited | 4 (17.39%) |
|  |  | The global human functioning in a standardized environment | 1 (4.35%) |
|  |  | The highest probable level of functioning that a person may reach in a given domain (correct) | 7 (30.43%) |
|  |  | The individual's ability to execute a task or an action | 8 (34.78%) |
|  |  | The lived experience of people in the actual context in which they live (correct) | 3 (13.04%) |
|  | **ICF.Q4. In which component of the ICF are prosthetic and orthotic devices classified** | Activities and participation | 10 (45.45%) |
|  |  | Body functions | 6 (27.27%) |
|  |  | Body structures | 4 (13.64%) |
|  |  | Environmental factors (correct) | 1 (4.55%) |
|  |  | Personal factors | 2 (9.09%) |
| **Use of ICF** | | | |
|  | **Do you use ICF?** | Yes | 13 (56.52%) |
|  |  | No | 10 (43.48%) |
|  | **Where do you use ICF?** | Clinic | 7 (53.85%) |
|  |  | Clinic Research | 1 (7.69%) |
|  |  | Clinic Teaching | 1 (7.69%) |
|  |  | Research | 3 (23.08%) |
|  |  | Teaching | 1 (7.69%) |
|  | **When did you learn about ICF?** | Bachelor’s Degree | 14 (56%) |
|  |  | CME/CPD Courses | 1 (4%) |
|  |  | Clinical Practice | 2 (8%) |
|  |  | Master or PhD | 4 (16%) |
|  |  | Specialization | 4 (16%) |
